# Zinc(II) Complexes of Amino Acids as New Active Ingredients for Anti-Acne Dermatological Preparations

**DOI:** 10.3390/ijms22041641

**Published:** 2021-02-06

**Authors:** Michał Abendrot, Elżbieta Płuciennik, Aleksandra Felczak, Katarzyna Zawadzka, Ewelina Piątczak, Piotr Nowaczyk, Urszula Kalinowska-Lis

**Affiliations:** 1 Department of Cosmetic Raw Materials Chemistry, Faculty of Pharmacy, Medical University of Lodz, Muszyńskiego 1, 90-151 Łódź, Poland; michal.abendrot@gmail.com; 2 Department of Molecular Carcinogenesis, Medical University of Lodz, Żeligowskiego 7/9, 90-752 Łódź, Poland; elzbieta.pluciennik@umed.lodz.pl; 3 Department of Industrial Microbiology and Biotechnology, Faculty of Biology and Environmental Protection, University of Lodz, 12/16 Banacha Street, 90-237 Łódź, Poland; aleksandra.felczak@biol.uni.lodz.pl (A.F.); katarzyna.zawadzka@biol.uni.lodz.pl (K.Z.); 4 Department of Biology and Pharmaceutical Botany, Faculty of Pharmacy, Medical University of Lodz, Muszyńskiego 1, 90-151 Łódź, Poland; ewelina.piatczak@umed.lodz.pl; 5 Department of Health Sciences, University of Opole, Katowicka 68, 45-069 Opole, Poland; piotr.nowczy@uni.opole.pl

**Keywords:** zinc(II) complexes, amino acids, acne, cytotoxicity, antibacterial activity, skin tolerance

## Abstract

Zinc compounds have a number of beneficial properties for the skin, including antimicrobial, sebostatic and demulcent activities. The aim of the study was to develop new anti-acne preparations containing zinc–amino acid complexes as active ingredients. Firstly, the cytotoxicity of the zinc complexes was evaluated against human skin fibroblasts (1BR.3.N cell line) and human epidermal keratinocyte cell lines, and their antimicrobial activity was determined against *Cutibacterium acnes*. Then, zinc complexes of glycine and histidine were selected to create original gel formulations. The stability (by measuring pH, density and viscosity), microbiological purity (referring to PN-EN ISO standards) and efficacy of the preservative system (according to Ph. Eur. 10 methodology) for the preparations were evaluated. Skin tolerance was determined in a group of 25 healthy volunteers by the patch test. The preparations containing zinc(II) complexes with glycine and histidine as active substances can be topically used in the treatment of acne skin due to their high antibacterial activity against *C. acnes* and low cytotoxicity for the skin cells. Dermatological recipes have been appropriately composed; no irritation or allergy was observed, and the preparations showed high microbiological purity and physicochemical stability.

## 1. Introduction

Zinc acts as cofactor for over 1000 biochemical reactions impacting the growth, differentiation and development of cells in different tissues [1]. It is an essential microelement and its proper skin content plays a key role in the prevention of many skin diseases [2]. Zinc-containing compounds are hence often used as active substances in dermatological products [3]. Most zinc-containing substances are exploited for their antimicrobial and anti-acne potential as they may represent effective safer alternatives for current treatments with systemic adverse effects [4,5]. Several mechanisms have been proposed for the effects of zinc in acne-affected skin, with the most significant being antimicrobial activity against *C. acnes* and lipase inhibition, reduction in sebum production [6], anti-inflammatory effects [6,7] and 5α-reductase inhibition [8].

Acne vulgaris is defined as an inflammation of the pilosebaceous units (hair follicles and sebaceous glands), manifested by the overproduction of sebum, keratinization disturbance, excessive multiplication of skin microflora and, finally, the formation of blackheads, inflammatory pimples, papules and scars. The pathogenesis of acne vulgaris is extremely complex and has not been fully described; however, it is believed that this disease is caused by the excessive production of androgen hormones, follicular hyperkeratinization and the colonization of hair follicles by bacteria, including strains of *Cutibacterium acnes* and *Staphylococcus epidermidis* [9,10].

*C. acnes* is a Gram-positive, anaerobic, polymorphic microorganism which is associated with the pathogenesis of acne vulgaris; it hence constitutes the typical target of antibiotic therapy. Antibiotic therapy of acne involves the use of drugs from the macrolide group, mainly erythromycin and clindamycin. However, the misuse of antibiotics, often combined with their prolonged use, has resulted in the evolution of bacterial resistance to traditional drugs. Such cases have already been noted in many countries of the European Union [10,11]. Therefore, it is essential to search for alternative substances that can be effective in acne treatment. The effect of zinc on acne has been well described since the first clinical trials on oral administration of zinc sulphate were conducted in 1977 [4]. Nowadays however, it is known that organic derivatives of zinc possess better bioavailability and tolerability than typical inorganic zinc salts [12,13,14]. Interestingly, it was shown that zinc absorption is increased by the addition of amino acids [15] and chelating peptides forming metal coordination complexes [16]. Moreover, zinc ions were found to reduce antibiotic resistance [17] and increase their topical absorption rate (as for erythromycin) [6]. In addition, the effects of zinc compounds in acne might exceed those for some antibiotic therapies [18]. Taking into account the growing antibiotic resistance problem [19,20], there is a need to identify new non-antibiotic solutions for acne treatment. Therefore, we have recently synthesized and fully characterized a number of new zinc complexes with amino acids and evaluated their antibacterial activity against a few selected pathogens [21].

When treating acne, it is essential to choose the correct cosmetic products as they constitute the essential foundation for everyday skin management [22]. The maximal concentration of zinc permitted in a cosmetic product in the EU is 1% [23]. Although various zinc amino acid salts can be used in cosmetic products, only zinc aspartate, zinc cysteinate, zinc glutamate and zinc glycinate have been included in the latest Cosmetic Ingredient Review (CIR) and in the cosmetic product EC Regulations [23,24]. Currently, a couple of these complexes have become an interest of animal feed [25,26] and the food industry [27,28], but their application in skin-applied products is scarce [24]. Although some reports suggest that such complexes might offer benefits against acne if taken orally [29], there are insufficient data regarding the efficacy and skin safety to consolidate their further use.

To fill this knowledge gap, the aim of this study was to carry out more extensive research to determine the potential application of selected zinc–amino acid complexes in dermatological products. Six zinc complexes of amino acids have been synthesized and characterized: ZnGlu (**1**), ZnGly (**2**), ZnHis (**3**), ZnPro (**4**), ZnMet (**5**) and ZnTrp (**6**) [21]. The present study evaluated their cytotoxicity against human skin fibroblasts (1BR.3.N cell line) and human epidermal keratinocytes (Neonatal, Millipore, co. SCCE020), as well as their antimicrobial activity against *Cutibacterium acnes* ATCC 6919 (formerly *Proprionibacterium acnes*). The two zinc complexes with the best parameters, ZnGly (**2**) and ZnHis (**3**), were selected and used to prepare stable gel formulas containing 0.2% zinc (i.e., 0.66% zinc glycine and 1.27% zinc histidine) and a non-zinc containing gel (placebo). The prepared gel preparations underwent six cycles of weekly physicochemical measurements of pH, density and viscosity, microbiological purity testing based on PN-EN ISO standards for cosmetic products, and efficacy of antimicrobial preservation (challenge test according to European Pharmacopoeia 10.0). Finally, to assess skin tolerance, all products were patch tested on healthy volunteers following the Jadassohn–Bloch method (modified by Rudzki) [30]. Therefore, this paper provides more detailed data for novel zinc complexes, substantiating their utilization in products for the management of acne-prone skin.

## 2. Results and Discussion

### 2.1. Evaluation of the Effect of Zinc Compounds on Keratinocyte and Fibroblast Viability

Novel substances must be subjected to a skin cytotoxicity evaluation before they can be used in a final product formula.

**Figure 1:**
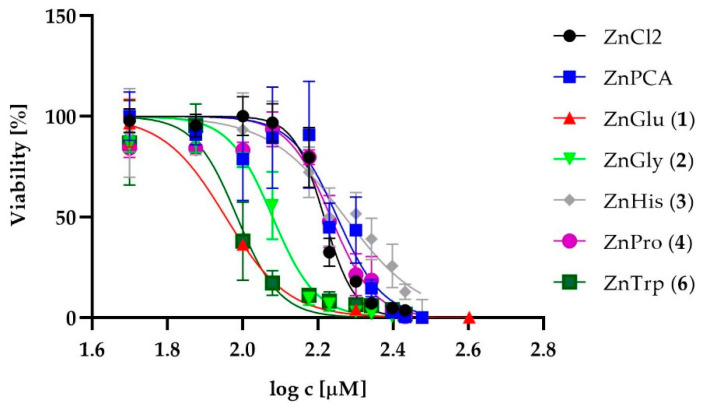
The normalized dose-response curves for the analyzed zinc complexes and reference compounds against fibroblasts.

**Figure 2:**
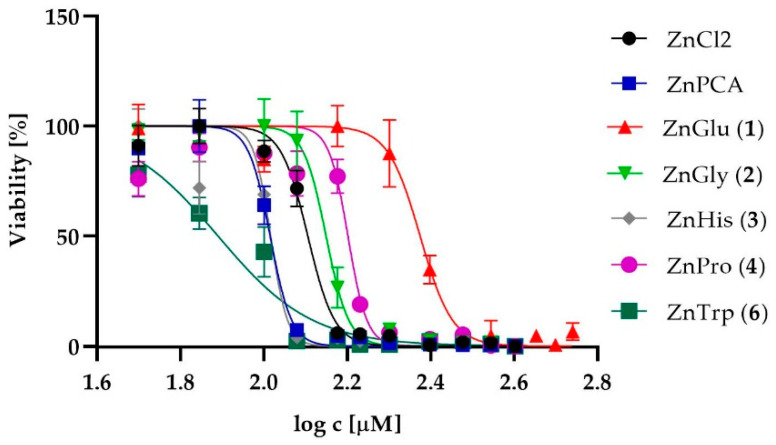
The normalized dose-response curves for the analyzed zinc complexes and reference compounds against keratinocytes

To assess the safety of their external use as active ingredients in dermatological preparations, the cytotoxicity of the zinc complexes, i.e., ZnGlu (**1**), ZnGly (**2**), ZnHis (**3**), ZnPro (**4**) and ZnTrp (**6**), was tested in vitro against human skin fibroblasts (1BR.3.N cell lines) and human epidermal keratinocytes, neonatal cells. ZnPCA (zinc 2-pirrolidone 5-carboxylate) and ZnCl_2_ were used as reference compounds. ZnMet (**5**) was not evaluated due to insufficient solubility. The normalized dose-response curves for analyzed zinc complexes and reference compounds for fibroblasts and keratinocytes are shown in Figure 1 and Figure 2, respectively.

The IC_50_ values for the zinc complexes against human skin fibroblasts ranged from 87.38 to 172.20 (µM) (Table 1). The complexes ZnHis (**3**) and ZnPro (**4**) were the least cytotoxic, with the IC_50_ values at the similar levels without statistical differences (at *p* ≤ 0.05) as the reference compound ZnPCA (171.73 µM). The IC_50_ values of complexes (**1**), (**2**) and (**6**) were significantly lower (at *p* ≤ 0.05) than both controls. The most cytotoxic towards fibroblasts were the complexes ZnGlu (**1**) and ZnTrp (**6**): their cytotoxicity was about twice that of the reference compounds.

Different sensitivity of human epidermal keratinocytes to the zinc complexes was observed (Table 1, Figure 2). The IC_50_ values of the complexes ZnGlu (**1**), ZnGly (**2**) and ZnPro (**4**) were significantly higher than both controls. However, the highest cytotoxic effect among all analyzed complexes was demonstrated by ZnTrp (**6**), but without statistical differences (at *p* ≤ 0.05) between the cytotoxicity of ZnHis (**3**) and ZnPCA. ZnHis (**3**) demonstrated equal cytotoxicity to the reference compound ZnPCA (102.50 µM).

Considering the cytotoxic activity of the complexes against skin cells, fibroblasts and keratinocytes, it could be concluded that the most favorable cytotoxicity profile was shown by the ZnPro (**4**), ZnHis (**3**) and ZnGly (**2**) complexes.

Previous studies have demonstrated that a concentration of ZnCl_2_ equal to 100 μM was not only non-toxic to HaCaT keratinocytes, but also increased cell proliferation and induced significant changes in the mRNA expression of NOTCH1, IL8 and cyclooxygenase-2 [31]. ZnCl_2_ was used as a reference substance in the present study; it demonstrated an IC_50_ value of 127 μM neonatal human epidermal keratinocytes.

ZnO nanoparticles (ZnO NP) demonstrated higher toxicity against keratinocytes than the equivalent concentration of ZnSO_4_. In addition, zinc chelators such as bovine serum albumin (BSA) and EDTA mitigated overall zinc toxicity [32]. In turn, the zinc histidine complex demonstrated significantly lower cytotoxic potential than ZnSO_4_ against normal human keratinocytes (NHK). Both derivatives exert stimulatory effects on natural NHK proliferation and differentiation; however, the zinc histidine derivative demonstrated a significantly greater proliferation enhancement effect than the inorganic salt, whereas zinc sulphate displayed a more pronounced influence on differentiation [13].

The toxicity of ZnCl_2_ against fibroblasts is well characterized, but only in a murine model: it exerts substantial toxicity against L929 fibroblast cultures, with an IC_50_ value of 0.928 μM [33]. Our present toxicity data indicate significantly lower toxicity against human skin fibroblasts, with an IC_50_ value of 162.65 μM.

Concerning the other metal complexes of amino acid derivatives, the toxicity of copper(II) complex with 5-hydroxytryptophan was tested on a few cell types, including a non-malignant human lung fibroblast (MRC-5) cell line, and showed no toxic effects at concentrations up to 100 µM [34].

### 2.2. Antimicrobial Activity against the Anaerobic Strain Cutibacterium acnes ATCC 6919

Zinc complexes have been previously demonstrated to have antibacterial activity against certain Gram-positive strains, namely *Staphylococcus aureus* ATCC 6538, *Staphylococcus epidermidis* ATCC 12228, and *Streptococcus pyogenes* ATCC 19615, and two Gram-negative bacteria, viz. *Escherichia coli* ATCC 25992 and *Pseudomonas aeruginosa* ATCC 27853 [21]. The present paper examines the activity of these complexes against a strain of *Cutibacterium acnes* ATCC 6919, an anaerobic bacterium.

Acne vulgaris, one of the most commonly diagnosed dermatological disorders, is a chronic disease that affects people of all ages, particularly teenagers. Although it is not a life-threatening disorder, it is a considerable therapeutic and psychological problem [4].

The present study determined the anti-acne properties of six newly-synthesized zinc complexes (**1**–**6**). The obtained results are presented in Table 2.

The anti-acne properties of the studied complexes were compared with those of ZnPCA, a compound commonly used in the treatment of various skin lesions. The highest antibacterial activity was expressed by the complexes ZnGly (**2**), ZnHis (**3**) and ZnMet (**5**), with minimum inhibitory concentration (MIC) values of 100, 125 and 125 mg/L, respectively. Interestingly, ZnGly (**2**) demonstrated almost twice the antibacterial activity of ZnPCA and showed good antimicrobial activity toward *S. epidermidis* [21]. It may therefore prove to be an effective weapon against the growth in drug resistance among cutaneous bacteria, and represent an alternative for conventional drug therapies.

Various compounds, such as essential oils and antimicrobial peptides originating from plants, as well as some retinoids, may show activity against *C. acnes* [10,11,35]. Miazga-Karska et al. [11] showed that the low molecular weight burdock peptide Br-p inhibited the growth of *C. acnes* at concentrations lower than 63 mg/L, depending on the strain tested. The peptide was also found to limit the growth of *S. aureus* and *S. epidermidis* at 250 and 500 mg/L, respectively. Additionally, Mustarichie et al. [36] revealed that the extract of cassava leaves exhibited antimicrobial activity against both *C. acnes* and *S. epidermidis*. The published data also indicate that non-antibiotic substances could reduce the numbers of *C. acnes*. Isotretinoin, which is a non-antimicrobial retinoid, can effectively reduce the multiplication of *C. acnes* [37].

There are a few mechanisms that explain the bactericidal activity of zinc compounds; however, different zinc derivatives could exert various modes of action. The mechanisms of action for ZnO NPs include: direct contact killing, the production of reactive oxygen species (ROS) and Zn^2+^ ions release. These effects can lead to the destruction of the bacterial membrane, disturbance of the respiration process of the cell, enzyme denaturation, and the inhibition of DNA replication, causing leakage of the cell content and its death [38,39]. The antibacterial mechanism for the majority of zinc derivatives seems to be dependent on the level of Zn(II) ions released. Achieving Zn^2+^ concentrations above 10^−4^ M was shown to have a toxic effect on prokaryotes by increasing the cell membrane permeability in a dose-dependent manner, as for zinc oxide and zinc gluconate [40]. On the other hand, some aspects of the antimicrobial effect of zinc complexes were explained by Overton’s concept of cell permeability, according to which the lipid cell membrane favors the passage of lipophilic substances [41]. The activity of Zn(II) complexes of Schiff base derivatives was elucidated based on chelation theory. This study showed that the polarity of Zn^2+^ was reduced by the chelation of Schiff bases that enhanced the lipophilicity of the zinc complexes. As a result, zinc chelate complexes can better penetrate the lipid membrane of microorganisms than corresponding free ligands [41,42,43].

### 2.3. Assesment of Antifungal Activity of Zinc Complexes (***1**–**6***)

Antifungal properties have been determined for both yeast *(Candida albicans* ATCC 10231, *Candida parapsylosis* ATCC 22019) and microscopic filamentous fungi (*Aspergillus flavus ATCC 9643*, *Aspergillus fumigatus* ATCC 204305). Zinc–amino acid complexes were tested in a concentration range of 0 to 500 mg/L. The conducted analyses showed that the addition of the mentioned complexes did not affect the growth or morphology of the tested strains. Therefore, no minimum fungicidal concentration (MFC) values were determined for the synthetized complexes.

### 2.4. Preparation of Gel Formulations: ZnGly Gel (**A**), ZnHis Gel (**B**) and Placebo Gel

The percentage concentration of ZnGly (**2**) in gel formulation (**A**) was 0.66%, while that of ZnHis (**3**) in (**B**) was 1.27% [44]. These formulations contained the same molar equivalent of zinc ions as in the 1% ZnPCA (PCA—pyrrolidone carboxylic acid). Thus, in one mole of 1% ZnPCA there is 0.2022 g of zinc ions. A more detailed composition of gel preparations is presented in Table 7 (Section 3. Materials and Methods). In addition to the active substances ZnGly (**2**) and ZnHis (**3**), the preparations contained the following ingredients: iota-carrageenan, which was used as a gelling agent; a mixture of phenoxyethanol: ethylhexylglycerin (9: 1 *w*/*w*) acting as a preservative of the preparations; and citric acid to adjust the pH of the preparations to the value 5.0–5.5.

It is established that the pH value on the skin’s surface ranges from 5.0 to 6.0 [45]. In addition, acne vulgaris patients tend to demonstrate higher facial skin pH values than age- and sex-matched controls without acne (5.09 ± 0.39 vs. 6.35 ± 1.30) [46]. Regarding our gel preparations, ZnGly gel (**A**) was pH 5.30±0.05, ZnHis gel (**B**) was pH 5.30 ± 0.05 and placebo gel was pH 5.36 ± 0.04.

### 2.5. Stability Assessment of Gel Formulations: ZnGly Gel (**A**), ZnHis Gel (**B**) and Placebo Gel

The stability of the prepared gel formulations was determined by testing their physicochemical parameters, including pH value, density and viscosity, once a week for six weeks. Additionally, organoleptic tests were performed to assess the physical appearance of the preparations, i.e., their color and odor. More detailed results are given in Table 3A–C with calculated standard deviations (n = 3) from the physicochemical evaluations.

Only slight changes in the measured parameters—pH, density and viscosity values—were observed (*p* ≤ 0.05), so they can be omitted in all products. All evaluated preparations (ZnGly gel (**A**), ZnHis gel (**B**) and the placebo gel) therefore appeared to remain stable at room temperature for at least six weeks. Additionally, no changes in color and odor were observed after six weeks of testing for any preparation.

### 2.6. Microbiological Tests of Gel Formulations: ZnGly Gel (**A**), ZnHis Gel (**B**) and Placebo Gel

Microbiological tests are required during the development of a dermatological preparation [23]: it has to fulfil the appropriate microbiological purity criteria, and the formula should prevent microbial growth during storage and use. As the antimicrobial efficacy may be influenced by the other constituents and not only the active substances, this test must be performed for every preparation separately. Additionally, the other antimicrobial properties of the examined active substances may be established as for anti-acne preparations.

The microbiological purity test was performed according to the PN-EN ISO standards for cosmetic products. The efficacy of antimicrobial preservation was confirmed according to the European Pharmacopoeia 10.0 (Ph. Eur. 10.0).

#### 2.6.1. Microbiological Purity Test

All three preparations complied with ISO microbiological purity standards. The tested preparations were deprived from the key skin pathogens *Pseudomonas aeruginosa, Escherichia coli, Staphylococcus aureus* and *Candida albicans.* The numbers of aerobic mesophilic microorganisms, yeast and molds were within established ISO limits (Table 4). The detailed results are given in the Supplementary Materials (Test reports S1–S3) for each tested preparation.

The microbiological purity test is a basic step which plays an essential role in assessing the quality of each product type; it is therefore frequently utilized, for example, as part of the product stability testing process. Although cosmetic products are not expected to be fully aseptic, the presence of any microorganisms should be established, as well as their number [47]. Furthermore, the microbiological purity of each batch of product should be established before it is launched onto the market [48]. This procedure also ensures that the results of further tests would not be disrupted when developing the product. If the microbiological purity standard of a sample preparation is confirmed, further antimicrobial protection tests and dermatological examinations can be performed to gain additional information about the product. In our study, the examined preparations fulfilled all seven microbiological purity criteria according to the established ISO standards for cosmetic products.

#### 2.6.2. Test for Efficacy of Antimicrobial Preservation (Challenge Test)

All tested preparations demonstrated effective antimicrobial preservation: the recommended efficacy protection criteria (A criteria) for cutaneous application preparations were in line with Ph. Eur. 10.0 standards (Table 5 and Table 6). No differences were observed between the three preparations, viz. ZnGly gel (**A**), ZnHis gel (**B**) and placebo gel, regarding the log reduction in any tested time period for any microbial strain type. Detailed results are presented in the Supplementary Materials (Test reports S4–S6). 

Antimicrobial activity must be evaluated for products that may demonstrate microbial growth during use or storage. This is particularly important for multiple use products, unless they possess self-preserving properties [49]. The vast majority of cosmetics are not single-use products, so antimicrobial activity efficacy must be determined to ensure consumer safety [50]. Such tests must be performed for each product separately to account for the addition of any further active or auxiliary substances. In the present study, the challenge test was performed according to the European Pharmacopea standard (Ph. Eur. 10.0), as this is a well-known international standard.

Separate tests were performed for each product, viz. ZnGly gel (**A**), ZnHis gel (**B**) and placebo gel, and all tested preparations demonstrated the highest possible level of antimicrobial preservation according to the Ph. Eur. 10.0 standard. As the preservative system, we used a 1% mixture of Phenoxyethanol (PE) with Ethylhexylglycerin (EHG) (9:1 *w*/*w*); although the used PE concentration was almost the maximal permitted by Regulation (EC) No. 1223/2009, Annex V, entry 29, the maximal concentration of EHG allowed in cosmetic products is not established [23]. We observed that the zinc-containing active substances (0.66% ZnGly and 1.27% ZnHis) did not influence the antimicrobial properties of the used preservative system: neither zinc complex appeared to interact negatively with the preservative system, nor did they demonstrate any additional antimicrobial enhancing properties. The preservative system in the examined preparations was so effective that maximal logarithmic microbial reduction was observed two days after microbial inoculation.

### 2.7. Dermatological Test (Patch Test) of Gel Formulations: ZnGly Gel (**A**), ZnHis Gel (**B**) and Placebo Gel

None of the tested products showed any irritating or allergenic properties, as indicated by a patch test conducted on a group of 25 healthy volunteers with a history of allergy. The detailed results for each examined product are presented in the Supplementary Materials (Test reports S7–S9).

Patch testing is recognized as a standard procedure for the assessment of irritant and sensitizing properties of a product [51]. Although the zinc bisglycinate complex is commonly used in dietary supplements and animal feed, its use in skin-applied products remains scarce [28].

Experiments conducted on animals indicate that some water-soluble zinc salts, especially aqueous solutions of inorganic zinc salts (1% ZnCl_2_ and 1% ZnSO_4_), may be skin irritants [52]. Furthermore, the Cosmetic Ingredient Review (CIR) underlines that any additional data concerning the safety of zinc salts utilized in cosmetic products would be favorable [24]. Our results provide new data showing that the gel preparations containing 0.66% ZnGly (**2**) and 1.27% ZnHis (**3**) demonstrate no irritating effects against the skin of patients with a history of allergy.

Although zinc derivatives are frequently used in a wide range of products, including cosmetics and pharmaceuticals, only a few cases of zinc salt allergy have been reported [52]. In addition, only a few positive results have been recorded of patch testing for zinc pyrithione [53,54]. Therefore, our present findings may provide the first such evidence that the amino acid-based complexes zinc-glycinate and zinc-histidine do not demonstrate skin sensitizing properties.

## 3. Materials and Methods

### 3.1. Materials and Reagents

The zinc(II) complexes of amino acids (**1**–**6**) were prepared according to a previously described procedure [21]. ZnPCA was purchased from Ajinomoto OmniChem and ZnCl_2_ from Merck (Darmstadt, Germany). Carrageenan (Satiagel™ VPC 508 P) was delivered from Univar Solutions (Warsaw, Poland). The preservative mixture (Phenoxyethanol, Ethylhexylglycerin, 9:1 *w*/*w*) was purchased from Schülke & Mayr (Norderstedt, Germany). Citric acid monohydrate was delivered by Brenntag (Kędzierzyn-Koźle, Poland). The water used for each sample preparation was demineralized and obtained by reverse osmosis.

### 3.2. Evaluation of the Effect of Zinc Compounds on Keratinocytes and Fibroblast Viability

The cytotoxicity test was conducted on a 1BR.3.N cell line (human skin fibroblasts, ECACC, co. 90020508) and human epidermal keratinocytes, neonatal (Millipore, co. SCCE020) purchased from Sigma-Aldrich (Merck, Darmstadt, Germany).

The 1BR.3.N cells were seeded 24 h before the experiments into 96-well plates at 10^4^ cells/well density, in EMEM (EBSS) culture medium supplemented with 2 mM glutamine, 1% nonessential amino acids (NEAA), 10% fetal bovine serum (FBS), and 1% antibiotic-antimycotic.

The same procedure was conducted for keratinocytes in EpiGRO™ human epidermal keratinocyte complete media with 6 mM l-glutamine, 0.405 EpiFactor P, 1.0 μM epinephrine, 0.5 ng/mL rh TGF-α, 100 ng/mL hydrocortisone hemisuccinate, 5 μg/mL rh insulin and apo-transferrin.

The cells were maintained at 37 °C and 5% CO_2_ in a humidified incubator. Next, the medium was changed into a starvation medium for the 1BR.3.N cell line (EpiGRO™ human epidermal keratinocyte complete media kit for keratinocytes) with a variable final concentration of five analyzed complexes (ZnGlu (**1**), ZnGly (**2**), ZnHis (**3**), ZnPro (**4**), ZnTrp (**6**)) and two controls, ZnPCA and ZnCl_2_. 

Stock solution for complex (**6**) was prepared in DMSO, and the remaining zinc compounds were dissolved in water. Zn(II) complex of methionine (**5**) was not detected because of insufficient solubility in DMSO and water. Final dilutions of the complexes were made in cell culture medium. Vehicle controls (both for 0.1% DMSO and water) were made, but no differences were observed with the untreated cells. After 24 h of incubation, the cytotoxicity was evaluated using the Presto Blue cell viability assay on a VICTOR™ X4 Multilabel Plate Reader (Perkin Elmer, London, UK). IC_50_ values were calculated using the GraphPad Prism 5.01 software tool (CDO-Group Sp. z o.o., Warsaw, Poland).

### 3.3. Evaluation of the Antibacterial Activity of Zinc Complexes (***1**–**6***)

The antimicrobial potential of zinc complexes against the anaerobic strain *Cutibacterium acnes* ATCC 6919 was tested by the microdilution method in Brucella broth supplemented with hemin, vitamin K1 and laked horse blood according to the CLSI document M11 (9th Edition) for antimicrobial susceptibility testing of anaerobic bacteria. Antimicrobial activity testing assays were conducted in 96-well cell culture plates in anaerobic conditions. The antimicrobial properties of zinc complexes were tested up to 500 mg/L. A bacterial inoculum prepared in reduced supplemented Brucella broth was added to each well to achieve a final density of 1 × 10^6^ CFU/mL. The bacterial suspension and dilutions of the tested compounds were combined in equal volumes to achieve the expected concentrations of zinc amino acid complexes. Samples as well as adequate abiotic and biotic controls were incubated for 48 h at 37 °C after being placed in 96-well cell culture plates in anaerobic jars, in which anaerobic conditions were formed using Anoxomat Mark II CTS (Mart Microbiology B.V., Drachten, the Netherlands). The minimum inhibitory concentration (MIC), expressed in mg/L, was determined as the lowest concentration of zinc amino acid complexes at which no growth of *C. acnes* was observed. Next, the Brucella agar plates were inoculated with 200 µL of *C. acnes* suspension taken from the wells in which no bacterial growth was observed. The Brucella agar plates were incubated for 48 h at 37 °C after placing the plates in anaerobic jars, in which anaerobic conditions were created.

### 3.4. Assessment of Antifungal Properties of Zinc Complexes (***1**–**6***)

The antifungal potential of zinc amino acid complexes was tested by the microdilution method in RPMI 1640 medium without bicarbonate, with glutamine and phenol red as a pH indicator according to CLSI guidelines M27 (4th Edition) and M38 (3th Edition) for broth dilution antifungal susceptibility testing of yeast and filamentous fungi, respectively. Assessment of antifungal activity was conducted on *C. albicans* ATCC 10231, *C. parapsylosis* ATCC 22019, *A. flavus* ATCC 9643 and *A. fumigatus* ATCC 204305 in 96-well cell culture plates. The antifungal activity of zinc amino acid complexes against yeast and filamentous fungi was assessed up to 500 mg/L. Final densities of yeast cells and fungi spores in each well were 2.5 × 10^3^ CFU/mL and 2.5 × 10^4^ CFU/mL, respectively. Samples containing zinc amino acid complexes as well as adequate biotic and abiotic controls were incubated for 48 h at 37 °C. After incubation, the minimum fungicidal concentration (MFC) was determined as the lowest concentration of the tested compounds that completely inhibited the growth of microorganisms.

### 3.5. Preparation of Gel Formulations: ZnGly Gel (**A**), ZnHis Gel (**B**) and Placebo Gel

The detailed composition of each prepared gel formulation is depicted in Table 7. The percentage concentrations of the active substances, i.e., ZnGly (**2**) and ZnHis (**3**), in gel formulations were 0.66% and 1.27%, respectively.

Each formula was prepared at room temperature. The carrageenan (2.0 g) was spread thoroughly into the water (70 mL) until the gel was formed. The mixture of phenoxyethanol: ethylhexylglycerin (9:1) (1.0 g) was added. The mixture was homogenized (7 000 RPM, 1 min/100 g). The proper zinc complex (0.66 g of ZnGly (**2**) and 1.27 g of ZnHis (**3**)) was added and mixed. While stirring, aqueous 2.5% citric acid solution (i.e., 0.52 g for placebo gel, 14.76 g for ZnGly gel (**A**) and 22.75 g for ZnHis gel (**B**)) was added to achieve a pH value between 5.0 and 5.5. The obtained gels were then homogenized (7 000 RPM, 2 min/100 g) and stored for seven days till the air bubbles disappeared.

### 3.6. Stability Assessment of Gel Formulations: ZnGly Gel (**A**), ZnHis Gel (**B**) and Placebo Gel

In order to assess the stability of prepared gel formulations, i.e., ZnGly gel (**A**), ZnHis gel (**B**) and placebo gel, their three physicochemical parameters, including pH, density and viscosity, were evaluated every week for six weeks. All samples were stored at room temperature (23.8 ± 0.6 °C).

#### 3.6.1. pH Testing

The pH values of the prepared formulations were determined using a calibrated Digital pH meter (Seven Compact meter, Mettler-Toledo, Warsaw, Poland) with the pH value accuracy (±)0.01. The measurement process was performed directly as all examined products were water-based gel formulas. Every measurement of pH of each formulation was triplicated and mean values were noted.

#### 3.6.2. Viscosity Measurements

The viscosity measurements of prepared gel formulas were performed using the Rotational Viscometer (model R ViscoLead ONE, Fungilab, Barcelona, Spain). Depending on the gel formula, various types of spindle and its rotation speed were determined and then utilized. The following set of spindles and rotation speeds (revolutions per minute, RPM) was established: “R2, 30 RPM” for placebo gel, “R4, 2 RPM” for Zinc Glycinate gel and “R5, 2 RPM” for Zinc Histidine gel. Every viscosity measurement of each formulation was triplicated and mean values were noted. Considering the gels were airlocked immediately after their preparation, the first measurement was performed after one week. All measured values in centipoise [cP] are depicted in Table 3A–C. 

#### 3.6.3. Density Measurements

The density measurements for the gel preparations were performed using the calibrated metal pycnometer (P1 model, POL-ZAF, Wroclaw, Poland, V = 49.969 cm^3^). Every measured density value of each formulation was triplicated and the mean values were noted. The gels were airlocked immediately after their preparation and the first measurement was performed after one week. All measured values in g/cm^3^ are depicted in Table 3A–C.

### 3.7. Microbiological Tests of Gel Formulations: ZnGly Gel (**A**), ZnHis Gel (**B**) and Placebo Gel

#### 3.7.1. Microbiological Purity Test

Detection of the presence of microorganisms in the cosmetic product, and their quantification, was performed in accordance with the current applicable ISO standards:

1. PN-EN ISO 21149:2017-07 Cosmetics. Microbiology. Enumeration and detection of aerobic mesophilic bacteria.
2. PN-EN ISO 16212:2017-08 Cosmetics. Microbiology. Enumeration of yeasts and molds.
3. PN-EN ISO 18415:2017-07 Cosmetics. Microbiology. Detection of mold spores.
4. PN-EN ISO 22717:2016-01 Cosmetics. Microbiology. Detection of *Pseudomonas aeruginosa*.
5. PN-EN ISO 21150:2016-01 Cosmetics. Microbiology. Detection of *Escherichia coli.*
6. PN-EN ISO 22718:2016-01 Cosmetics. Microbiology. Detection of *Staphylococcus aureus*.
7. PN-EN ISO 18416:2016-01 Cosmetics. Microbiology. Detection of *Candida albicans.*

The research procedure included three separate phases: detection of the presence of the aforementioned microorganisms (1) and determination of the number of microorganisms (2 and 3):

1. Preparation of the suspension and preliminary multiplication on non-selective propagation broth—the decimal dilution suspension (the mixture of 1 g/mL sample and 9 mL Eugon LT100 broth) was mixed well and incubated under aerobic conditions. Preliminary multiplication on non-selective propagation broth was performed at a temperature range of 32.5 ± 2.5 °C for at least 20 h for aerobic mesophilic bacteria, or at least 20 h but not more than 72 h for *C. albicans, E. coli, P. aeruginosa* and *S. aureus*.
2. Cultivation and isolation on a selective agar medium (or non-selective in the case of aerobic mesophilic bacteria)—the samples were incubated aerobically at 32.5 ± 2.5 °C for 24–48 h (*C. albicans, E. coli, P. aeruginosa, S. aureus*) or 48–72 h (aerobic mesophilic bacteria, molds and yeasts) on medium TSA (aerobic mesophilic bacteria), SDA with chloramphenicol (*C. albican*s), MacConkey agar (*E. coli*), medium with cetrimide (*P. aeruginosa*), and Baird-Parker agar (*S. aureus*). For the determination of the number of microorganisms, after the incubation step, the colonies grown on the plate were counted and the number of microorganisms was determined (CFU/1 g (mL)).
3. Identification tests—performed using RapID biochemical identification tests (Remel, England), using conventional and chromogenic substrates to identify species of bacteria and fungi.

#### 3.7.2. Test for Efficacy of Antimicrobial Preservation (Challenge Test)

The effectiveness of antimicrobial protection was determined in accordance with the European Pharmacopoeia 10.0 (Ph. Eur. 10.0). The preparation demonstrates appropriate preservative properties if there is a significant decrease, or no increase, in the number of viable microbial cells in the inoculated preparation under the test conditions, after the specified time and at the specified temperature.

- Test Microorganisms

With the reference to Ph. Eur. 10.0., the preservation efficacy test of the dermal product was performed with inoculation of the preparation with the standardized following microorganisms: *Pseudomonas aeruginosa* ATCC 9027, *Staphylococcus aureus* ATCC 6538, *Escherichia coli* ATCC 8739, *Candida albicans* ATCC 10231 and *Aspergillus brasiliensis* ATCC 16404. Each microbial strain was tested on the preparation separately.

- Preparation of Inoculum

Each inoculum was prepared from a fresh culture of the aforementioned microorganisms. The individual cultures were incubated at the appropriate temperature for the appropriate time according to the indications of Ph. Eur. 10.0. The obtained microorganism suspensions had the following densities: for bacteria—10^7^–10^8^ CFU/mL, *C. albicans*—10^6^–10^7^ CFU/mL, *A. brasiliensis*—10^6^–10^7^ CFU/mL.

- Method Description

The study began with an assessment of the effectiveness of the neutralizer, followed by an evaluation of the preservation efficiency of the preparation intended for cutaneous application.

The study was performed separately for each test sample and test microorganism. The test sample (20 g) was infected with the strain (0.2 mL) and stored at room temperature (20–25 °C). The detailed concentrations utilized for each strain, complying with the Ph. Eur. 10.0. criteria, are presented in the Supplementary Materials (Test reports S4–S6). At specified time intervals (Table 5), 1 g of sample was taken into 9 mL of neutralizer, thoroughly mixed and incubated (bacteria and *C. albicans*—32.5 ± 2.5 °C for 48–72 h, *A. brasiliensis* 22.5 ± 2.5 °C for 3–5 days). After the incubation period, the grown colonies were counted; the degree of log reduction was then determined and compared to the Ph. Eur. 10.0. criteria (Table 5—acceptance criteria).

In the antimicrobial preservation efficiency test, the number of surviving microorganisms was determined at specified time intervals over a 28-day period. For each microbial strain, the log reduction index was calculated as the number of viable microorganisms against the value obtained for the inoculum; this value was compared with the criteria defined by Ph. Eur. 10.0. Detailed information of the criteria A and B is described in Table 5. The detailed results of each strain reduction over time, as indicated by the Ph. Eur. 10.0. criteria, are presented in the Supplementary Materials (Test reports S4–S6).

- Acceptance Criteria

The criteria for the evaluation of antimicrobial activity are presented in Table 5 (Section 2.6.2) and refer to the log reduction in each microorganism type.

The Ph. Eur. 10.0 expresses criteria A as recommended antimicrobial protection efficacy, implicating no additional preservatives are required. However, the antimicrobial protection efficacy level “B” might be accepted only in justified cases: for instance, if the A criterion cannot be achieved due to a significant risk of adverse reactions caused by the additional amounts of preservatives.

### 3.8. Dermatological Test (Patch Test) of Gel Formulations: ZnGly Gel (**A**), ZnHis Gel (**B**) and Placebo Gel

The examination was conducted according to the Jadassohn–Bloch method (modified by Rudzki) on a group of 25 healthy volunteers with a history of skin allergy [30]. All testing was performed under the supervision of a dermatologist. The main inclusion criteria were healthy females and males aged 18–65 years; phenotype: I–IV in the Fitzpatrick scale, caucasian skin type. The volunteers gave their voluntary consent to participate in the study. Their skin at the test site was normal, without any signs of irritation, allergy or any other pathological changes. 

The tested product was applied to filter paper in Finn Chambers. The whole patch was then applied to the arm skin of a volunteer and removed after 48 h. The first reading was performed 15–20 min after patch removal, and subsequently after 72 h.

The test result was interpreted according to the scale recommended by the International Contact Dermatitis Research Group (ICDRG) [51].

### 3.9. Statistical Analysis

Each experiment was performed in triplicate and repeated at least three times. All data are presented as means ± standard deviation (SD). The statistical significance was determined using Tukey’s HSD test (for comparison of physicochemical parameters) or the Kruskal–Wallis test (for comparison of cytotoxicity). Differences between means were considered significant at *p* ≤ 0.05. All the statistical analyses were calculated using Statistica software (STATSoft, Cracow, Poland, version 13.1, 2016).

## 4. Conclusions

Among the studied zinc complexes with amino acids, two of them, i.e., Zn complexes with glycine (**2**) and histidine (**3**), may constitute new active components of anti-acne preparations for topical treatment. The compounds ZnGly (**2**) and ZnHis (**3**) were selected because of their low in vitro cytotoxic potential for skin cells, both keratinocytes and fibroblasts, and high activity against *Cutibacterium acnes*.

The developed formulations: ZnGly gel (**A**) and ZnHis gel (**B**), based on the selected zinc complexes, were proven to be physicochemically stable and demonstrated high microbiological purity, with a well-selected preservative system and without any irritation or allergies in people with sensitive skin.

Therefore, the tested preparations (**A**) and (**B**) appear to have great potential for patients with symptoms of acne vulgaris. However, before they can be introduced to the market, further in vivo application studies with patients are needed to confirm their anti-acne effects.

In the next step of the research, it would be interesting to verify whether the active substances could play additional preservative roles in the preparations. This stage would require the preparation of new formulations without a preservative system and their further detailed testing. Obtaining preparations with such a simplified composition would be valuable, especially in terms of their use in the treatment of allergic skin prone to irritation.

## Figures and Tables

**Table 1:** The IC_50_ values (mean ± SD) for all analyzed zinc complexes for human fibroblasts and keratinocytes. The IC_50_ was calculated using the Hill Equation after the viable cell number was normalized relative to untreated cells.

	IC_50_ Values (Mean ± SD)	
Analyzed Complexes	Fibroblasts	Keratinocytes
ZnGlu (**1**)	87.38 ± 6.01 ^c^*	231.17 ± 15.94 ^d^
ZnGly (**2**)	119.85 ± 4.61 ^b^	138.20 ± 8.11 ^be^
ZnHis (**3**)	171.48 ± 17.02 ^a^	102.00 ± 3.76 ^ac^
ZnPro (**4**)	172.20 ± 4.00 ^a^	157.20 ± 8.19 ^b^
ZnMet (**5**)	-	-
ZnTrp (**6**)	94.88 ± 8.41 ^c^	85.66 ± 14.28 ^c^
ZnPCA **	171.73 ± 9.69 ^a^	102.50 ± 2.07 ^ac^
ZnCl_2_ **	162.65 ± 4.10 ^a^	126.97 ± 4.32 ^ae^

- not detected because of too little solubility in culture medium; * means signed with the same letter within each column are not significantly different (Kruskal–Wallis test; *p* ≤ 0.05); ** reference compounds (ZnPCA: zinc 2-pirrolidone 5-carboxylate).

**Table 2:** Antimicrobial activity against *C. acnes* of zinc complexes of amino acids (**1**–**6**).

Compound	MIC Value [mg/L]
ZnGlu (**1**)	250
ZnGly (**2**)	100
ZnHis (**3**)	125
ZnPro (**4**)	250
ZnMet (**5**)	125
ZnTrp (**6**)	175
ZnPCA *	175

* reference compound (zinc 2-pirrolidone 5-carboxylate).

**Table 3:** (**A**) Physicochemical evaluations of Zn-Glycine gel formulation; (**B**) Physicochemical evaluations of Zn-Histidine gel formulation; (**C**) Physicochemical evaluations of placebo gel formulation.

**(A)**				
**Stability Testing Week**	**ZnGly** **Gel**			
	**Color, Odor**	pH *	Density * [g/cm^3^]	Viscosity * [cP], (R4, 2 RPM)
0	slightly yellowish, no odor	5.35 ± 0.01 ^a**^	-	-
1	no changes	5.35 ± 0.01 ^a^	1.0046 ± 0.0001 ^d^	79,478 ± 1376 ^a,b^
2	no changes	5.34 ± 0.01 ^a^	1.0045 ± 0.0001 ^a^	82,332 ± 1094 ^b^
3	no changes	5.27 ± 0.02 ^b,c^	1.0044 ± 0.0001 ^a^	77,658 ± 1323 ^a^
4	no changes	5.26 ± 0.02 ^b^	1.0032 ± 0.0001 ^b^	77,233 ± 1276 ^a^
5	no changes	5.30 ± 0.01 ^c^	1.0044 ± 0.0001 ^a^	76,964 ± 1354 ^a^
6	no changes	5.25 ± 0.02 ^b^	1.0042 ± 0.0001 ^c^	77,796 ± 1083 ^a^
**(B)**				
**Stability Testing Week**	**ZnHis** **Gel**			
	**Color, Odor**	pH *	Density * [g/cm^3^]	Viscosity * [cP], (R5, 2 RPM)
0	slightly yellowish, no odor	5.35 ± 0.02 ^a**^	-	-
1	no changes	5.36 ± 0.01 ^a^	0.9788 ± 0.0001 ^a^	157,724 ± 2742 ^a^
2	no changes	5.35 ± 0.01 ^a^	0.9836 ± 0.0001 ^b^	154,249 ± 2866 ^a^
3	no changes	5.35 ± 0.01 ^a^	0.9786 ± 0.0001 ^c^	152,138 ± 2435 ^a^
4	no changes	5.33 ± 0.02 ^a^	0.9894 ± 0.0001 ^d^	154,403 ± 2009 ^a^
5	no changes	5.40 ± 0.01 ^b^	0.9928 ± 0.0001 ^e^	153,519 ± 2289 ^a^
6	no changes	5.40 ± 0.01 ^b^	0.9872 ± 0.0001 ^f^	155,302 ± 2162 ^a^
**(C)**				
**Stability Testing Week**	**Placebo Gel**			
	**Color, Odor**	pH *	Density * [g/cm^3^]	Viscosity * [cP], (R2, 30 RPM)
0	slightly yellowish, no odor	5.31 ± 0.02 ^a**^	-	-
1	no changes	5.35 ± 0.01 ^b^	1.0136 ± 0.0001 ^a^	971.7 ± 14 ^a^
2	no changes	5.32 ± 0.01 ^b^	1.0136 ± 0.0001 ^a^	1034.6 ± 13 ^b,c^
3	no changes	5.25 ± 0.02 ^c^	1.0136 ± 0.0001 ^a^	1089.7 ± 15 ^d^
4	no changes	5.27 ± 0.02 ^c^	1.0118 ± 0.0001 ^b^	1051.2 ± 12 ^c^
5	no changes	5.30 ± 0.02 ^a^	1.0116 ± 0.0001 ^b^	979.6 ± 12 ^a^
6	no changes	5.29 ± 0.02 ^a,c^	1.0106 ± 0.0001 ^c^	995.2 ± 11 ^a^

* Values expressed as mean ± SD of triplicate measurements (n = 3). ** Means indicated with the same letter within each column are not statistically different (Tukey’s test; *p* ≤ 0.05).

**Table 4:** Microbiological purity test of gel formulations.

Parameter	PN-EN ISO Requirements	ZnGly Gel (A)	ZnHis Gel (B)	Placebo Gel
Enumeration of aerobic mesophilic microorganisms	1 × 10^3^ CFU/1 g/1 mL	<1 × 10 ^1^ CFU/1 g/1 mL	<1 × 10^1^ CFU/1 g/1 mL	<1 × 10^1^ CFU/1 g/1 mL
Enumeration of yeast and mold	1 × 10^3^ CFU/1 g/1 mL	<1 × 10 ^1^ CFU/1 g/1 mL	<1 × 10^1^ CFU/1 g/1 mL	<1 × 10^1^ CFU/1 g/1 mL
Detection of mold spores	Absence in 1 g/1 mL	Absence in 1 g/1 mL	Absence in 1 g/1 mL	Absence in 1 g/1 mL
Detection of *Pseudomonas aeruginosa*	Absence in 1 g/1 mL	Absence in 1 g/1 mL	Absence in 1 g/1 mL	Absence in 1 g/1 mL
Detection of *Escherichia coli*	Absence in 1 g/1 mL	Absence in 1 g/1 mL	Absence in 1 g/1 mL	Absence in 1 g/1 mL
Detection of *Staphylococcus aureus*	Absence in 1 g/1 mL	Absence in 1 g/1 mL	Absence in 1 g/1 mL	Absence in 1 g/1 mL
Detection of *Candida albicans*	Absence in 1 g/1 mL	Absence in 1 g/1 mL	Absence in 1 g/1 mL	Absence in 1 g/1 mL

**Table 5:** The European Pharmacopoeia 10.0 criteria for efficacy of antimicrobial preservation.

Ph. Eur. 10.0. Requirements (Logarithm of Reduction in Time T_x_)								
Microorganisms	Bacteria *					Fungi **		
Sampling Time	T_2_	T_7_	T_14_	T_28_	T_2_	T_7_	T_14_	T_28_
Criterion A	≥2	≥3	-	≥3 i NI	-	-	≥2	≥2 NI
Criterion B	-	-	≥3	≥3 i NI	-	-	≥1	≥1 i NI

T_x_—time of performing measurements where “x” refers to the number of days; NI—the number of viable microorganisms does not increase compared to the previous reading; * *Pseudomonas aeruginosa* ATCC 9027, *Staphylococcus aureus* ATCC 6538, *Escherichia coli* ATCC 8739; ** *Candida albicans* ATCC 10231, *Aspergillus brasiliensis* ATCC 16404.

**Table 6:** The obtained results from the efficacy of antimicrobial preservation test.

Ph. Eur. 10.0. Requirements	Final Sample Results		
	ZnGly Gel (A),	ZnHis Gel (B)	Placebo Gel
Criterion A	+	+	+
Criterion B	N/A	N/A	N/A

+—meets criterion A according to Table 5; N/A—not applicable.

**Table 7:** Detailed composition of gel formulations.

Components	ZnGly Gel (A)	ZnHis Gel (B)	Placebo Gel
Demineralized water	to 100 g	to 100 g	to 100 g
Iota-Carrageenan	2 g	2 g	2 g
Phenoxyethanol: Ethylhexylglycerin (9:1)	1 g	1 g	1 g
ZnGly (**2**)	0.66 g	N/A	N/A
ZnHis (**3**)	N/A	1.27 g	N/A
2.5% Citric Acid	14.76 g	22.75 g	0.52 g

N/A—not added.

## Data Availability

Data is contained within the article.

## Supplementary Materials

The following are available online at https://www.mdpi.com/1422-0067/22/4/1641/s1, Test report S1: microbiological purity test for ZnGly gel (**A**), Test report S2: microbiological purity test for ZnHis gel (**B**), Test report S3: microbiological purity test for placebo gel, Test report S4: challenge test for ZnGly gel (**A**), Test report S5: challenge test for ZnHis gel (**B**), Test report S6: challenge test for placebo gel, Test report S7: dermatological test (Patch test) for ZnGly gel (**A**), Test report S8: dermatological test (Patch test) for ZnHis gel (**B**), Test report S9: dermatological test (Patch test) for placebo gel.
