# Supplementary material for: Zinc(II) Complexes of Amino Acids as New Active Ingredients for Anti-Acne Dermatological Preparations
Source: Int J Mol Sci. 2021 Feb 6;22(4):1641. doi: 10.3390/ijms22041641 (PMC7915519; doi:10.3390/ijms22041641)
Supplement: Supplementary file 1 [file ijms-22-01641-s001.zip › ijms-1083706 suppl/Test report S2 - microbiological purity test for ZnHis gel (B).pdf]

dr Nowaczyk Centrum Badań i Innowacji Sp. z o.o. Sp.K.
ul. Żmigrodzka 81-83, lok. 205, 51-130 Wrocław, Poland
tel. +48 509 387 307 e-mail: biuro@drnowaczyk.com
www.drnowaczyk.com

Laboratorium badawcze zgodne z normą ISO / IEC 17025

Testing laboratory according to the standard ISO / IEC 17025

Sprawozdanie z badań, nr : / *Test report, no :* 0055 - MK / A / 09 / 20 rev. 01

(zgodnie z / *acc. to* LAB-05.00.02, rev.03)

BADANIE MIKROBIOLOGICZNE / KOSMETYKI / *MICROBIOLOGICAL TEST / COSMETICS*

Zleceniodawca : / *Customer:*
Zakład Chemii Surowców Kosmetycznych
Uniwersytet Medyczny w Łodzi
ul. Muszyńskiego 1
91-151 Łódź

Nazwa próbki: / *Name of sample:* Próbka: / Sample of : żel ŻDD_Zn-His_2.5%_1_16.07.20

Numer zlecenia: / *Protocol-order No.:* 0014 - Z / 09 / 20

Metoda pobrania próbki (5): / *Method of sampling (5) :* Nie podano / *Not given* rev.: (N)

Próbka pobrane przez: / *Sample collected by:* Klienta / *Customer*

Postępowanie z próbką wg: / *Handling of sample acc to::* LAB-05.00.00 rev.: 01

Stan i ilość próbki: / *Sample evaluation & quantity:* Bez zastrzeżeń / *Correct*

Data przyjęcia próbki: / *Date of receiving sample:* 02.09.2020

Numer próbki: / *Sample No.:* 0058 - P / 09 / 20

Data rozpoczęcia badania: / *Date of analysis start:* 02.09.2020

Numer analizy : / *Analysis No.:* 0055 - MK / A / 09 / 20

Data zakończenie badania: / *Date of of analysis end:* 08.09.2020

Wyniki badania odnoszą się wyłącznie do badanego obiektu / *Results are connected only with samples which were analysed*

L.p. / *No.*	Parametr / *Parameter*	Metoda / *Method*	Kryterium / *Requirements*		(5)	Wynik / *Result* [Jednostka/*Unit*]	U (6)
1.	Ogólna liczba drobnoustrojów tlenowych / Enumeration of aerobic mesophilic microorganisms	PN-EN ISO 21149:2017-07	1 x 10^3 CFU / 1g/1ml	(3)	(A)	**< 1 x 10^1** CFU / 1g/1ml	N/A
2.	Ogólna liczba pleśni i drożdży / Enumeration of yeast and mould	PN-EN ISO 16212:2017-08	1 x 10^3 CFU / 1g/1ml	(3)	(A)	**< 1 x 10^1** CFU / 1g/1ml	N/A
3.	Obecność zarodników pleśni / Detection of mold spores	PN-EN ISO 18415:2017-07	Nieobecne w 1g/1ml / *Absence in 1g/1ml*	(3)	(A)	Nieobecne w 1g/1ml / *Absence in 1g/1ml*	N/A
4.	Obecność *Pseudomonas aeruginosa* / Detection of *Pseudomonas aeruginosa*	PN-EN ISO 22717:2016-01	Nieobecne w 1g/1ml / *Absence in 1g/1ml*	(3)	(A)	Nieobecne w 1g/1ml / *Absence in 1g/1ml*	N/A
5.	Obecność *Escherichia coli* / Detection of *Escherichia coli*	PN-EN ISO 21150:2016-01	Nieobecne w 1g/1ml / *Absence in 1g/1ml*	(3)	(A)	Nieobecne w 1g/1ml / *Absence in 1g/1ml*	N/A
6.	Obecność *Staphylococcus aureus* / Detection of *Staphylococcus aureus*	PN-EN ISO 22718:2016-01	Nieobecne w 1g/1ml / *Absence in 1g/1ml*	(3)	(A)	Nieobecne w 1g/1ml / *Absence in 1g/1ml*	N/A
7.	Obecność *Candida albicans* / Detection of *Candida albicans*	PN-EN ISO 18416:2016-01	Nieobecne w 1g/1ml / *Absence in 1g/1ml*	(3)	(A)	Nieobecne w 1g/1ml / *Absence in 1g/1ml*	N/A

Legenda:

(1) - wyniki poniżej zakresu metody (<), dla wyniku poniżej zakresu metody (<) niepewności nie podaje się
(2) - wyniki z niepewnościa rozszerzoną - współczynnik rozszerzenia k=2; poziom ufności 95% nie obejmuje niepewności w obszarze pobierania próbki;
(3) - zgodnie z wewnętrznymi wymaganiami Klienta
(4) - badanie podzlecone
(5) - badanie/pobór próbki oznaczone symbolem (A) - badanie/pobór próbki planowane do akredytacji w 2020 roku; symbolem (N) - badanie/pobór próbki nieplanowane do akredytacji w 2020 roku
(6) - U - Niepewność pomiaru
N/A - nie dotyczy

Legend:

(1) - result is below the range of method (<), for the result below the range of method (<) uncertainty is not given
(2) - result with expanded uncenertanty - the coverage factor k=2, confidence level 95% does not include uncertainties in the sample;
(3) - according to internal Customer requirements
(4) - sub-contracted test
(5) - parameter (A) method / sampling planned for accreditation in 2020 ; parameter (N) - method / sampling not planned for accreditation in 2020
(6) - U - Uncertainty of measurement
N/A - not applicable

Ocena zgodności z wymaganiami / ***Conformity of assesment with the requirements :***

Badana próbka: / *Tested sample:* żel ŻDD_Zn-His_2.5%_1_16.07.20 odpowiada / *comply* wymaganiom określonym przez Klienta oraz **PN-EN ISO 17516:2014-01.** / *specified requirements of Customer and* ***PN-EN ISO 17516:2014-01.***

UWAGI DO WYNIKU: / *COMMENTS TO THE RESULT*
Ocena zgodności. Zasada podejmowania decyzji nr 3 "zasada wskazana przez Klienta". Uwzględnienie kryterów akceptacji wynikajacych z normy PN-EN ISO 17516:2014-01, bez uwzględnienia niepewności pomiaru.
Conformity assessment. Decision rule no. 3 "principle indicated by the client". Taking into account the acceptance criteria resulting from the PN-EN ISO 17516:2014-01 standard, without taking into account the measurement uncertainty.

COMMENTS: Brak / *No comments*

Autoryzował : / ***Authorized :*** 08.09.2020 (data / *date*) — dr Nowaczyk: 003 (Nr pracownika / *Employee no*) — kod QR /*QR code* — Kierownik Pracowni Mikrobiologii i Fizyko-Chemii (kwalifikowany podpis elektroniczny) * / *Microbiological & Physico-Chemical Laboratory Manager (qualified electronic signature) ** (funkcja / *position*)

(*) Bezpieczny podpis elektroniczny weryfikowany przy pomocy certyfikatu kwalifikowanego jest równoważny pod względem skutków prawnych z podpisem własnoręcznym. Oryginalene sprawozdanie z badań są wydawane w formie elektronicznej z rozszerzeniem "*-sig.pdf" lub "*_signed.pdf". W związku z tym wszystkie wydruki, o ile nie są potwierdzone za zgodność z oryginałem, są kopiami.

(*) Secure electronic signature verified by a qualified certificate is equivalent in terms of legal consequences handwritten signature. The original test report is issued in electronic form with the extension " -sig.pdf" lub "*-signed.pdf". Therefore, all prints, if not confirmed as being true to the original, are copies.*

Badania wykonywane w dr Nowaczyk Centrum Badań i Innowacji Sp. z o.o.Sp.K. są prowadzone zgodnie ze standardowymi metodami badawczymi. Uzyskane wyniki są zgodne z przyjętymi międzynarodowymi standardami, chyba że zostały ustalone inne szczegółowe wymagania, zatwierdzone na piśmie przez dr Nowaczyk Centrum Badań i Innowacji Sp. z o.o.Sp.K. oraz Klienta, przed rozpoczęciem badania. Wyniki badań odnoszą się wyłącznie do przebadanych próbek, w określonych warunkach i przy użyciu metod badawczych i procedur uzgodnionych wcześniej z Klientem. Obowiazkiem Klienta jest zapewnienie, że każda jednostka produktu i opakowania jest wyprodukowana w sposób identyczny. dr Nowaczyk Centrum Badań i Innowacji Sp. z o.o.Sp.K. nie ponosi odpowiedzialności za jakiekolwiek wnioski, interpretacje i uogólnienia wykonane przez Klienta z wydanych przez dr Nowaczyk Centrum Badań i Innowacji Sp.z o.o.Sp.K. sprawozdań z badań. Niniejsze Sprawozdanie z Badania jest poufną własnością Klienta. Bez pisemnej zgody dr Nowaczyk Centrum Badań i Innowacji Sp. z o.o.Sp.K. sprawozdanie z badania nie może być powielane inaczej jak tylko w całości.

Testing at dr Nowaczyk Centrum Badań i Innowacji Sp.z o.o.Sp.K. is performed according to standard test procedures. Reported test results are accurate to generally accepted international standards, unless specific requirements have been agreed to in writing by dr Nowaczyk Centrum Badań i Innowacji Sp.z o.o.Sp.K. and Customer prior to the test commencing. Our reports apply only to the specific samples tested, under stated conditions and using test methods and routines agreed in advance with our customers. It is the manufacturer's responsibility to assure that additional production units of product and package are manufactured identically. dr Nowaczyk Centrum Badań i Innowacji Sp.z o.o.Sp.K. shall have no liability for any deductions, inferences of generalisations drawn by the Customers or others from dr Nowaczyk Centrum Badań i Innowacji's issued reports. This report is the confidential property of dr Nowaczyk Centrum Badań i Innowacji Sp.z o.o.Sp.K. and its Customer. Extracts from the test report shall not be reproduced, except in full, and not without prior written approval of dr Nowaczyk Centrum Badań i Innowacji Sp.z o.o.Sp.K.

Sprawozdanie z badania zostało sporządzone w jednym egzemplarzu. / *Test Report prepared in single copy.*
Oryginał nr 1 : Klient / *Oryginal copy No. 1 : Customer*
Kopia egzemplarza nr 1 : Archiwum / *Duplicate of copy No. 1 : Archive*
