# Supplementary material for: Zinc(II) Complexes of Amino Acids as New Active Ingredients for Anti-Acne Dermatological Preparations
Source: Int J Mol Sci. 2021 Feb 6;22(4):1641. doi: 10.3390/ijms22041641 (PMC7915519; doi:10.3390/ijms22041641)
Supplement: Supplementary file 1 [file ijms-22-01641-s001.zip › ijms-1083706 suppl/Test report S6 - challange test for placebo gel.pdf]

dr Nowaczyk Centrum Badań i Innowacji Sp. z o.o. Sp.K.
ul. Żmigrodzka 81-83, lok. 205, 51-130 Wrocław, Poland
tel. +48 509 387 307e-mail: biuro@drnowaczyk.com
www.drnowaczyk.com

Laboratorium badawcze
zgodne z normą ISO / IEC 17025

Testing laboratory
according to the standard ISO / IEC 17025

Sprawozdanie z badań, nr : *Test report, no :* 0001 - MB / A / 09 / 20 rev. 04

(zgodnie z / *acc. to* LAB-05.00.03, rev.03)

TEST KONSERWACJI / KOSMETYKI / *CHALLENGE TEST / COSMETICS*

Zleceniodawca : *Customer:* Zakład Chemii Surowców Kosmetycznych
Uniwersytet Medyczny w Łodzi
ul. Muszyńskiego 1
91-151 Łódź

Nazwa próbki: *Name of sample:* Próbka: Sample of : żel ŻDD_2.5%_1_10.06.20

Metoda badawcza : *Test Method :* Ph. Eur. 10.0 , <5.1.3>

Numer zlecenia: *Protocol-order No.:* 0014 - Z / 09 /20

Metoda pobrania próbki (5)**:** *Method of sampling (5) :* Nie podano / *Not given* rev.: (N)

Próbka pobrane przez: *Sample collected by:* Klienta / *Customer*

Postępowanie z próbką wg: *Handling of sample acc to::* LAB-05.00.00 rev.: 01

Stan i ilość próbki: *Sample evaluation & quantity:* Bez zastrzeżeń / *Correct*

Data przyjęcia próbki: *Date of receiving sample:* 02.09.2020

Numer próbki: *Sample No.:* 0056 - P / 09/ 20

Data rozpoczęcia badania: *Date of analysis start:* 02.09.2020

Numer analizy : *Analysis No.:* 0001 - MB / A / 09 / 20

Data zakończenie badania: *Date of of analysis end:* 12.10.2020

Układ konserwujący : *Preservation System :* Euxyl PE 9010 1%

Wyniki badania odnoszą się wyłącznie do badanego obiektu / ***Results are connected only with samples which were analysed***

Neutralizator : Bulion Eugon LT 100, skład: pankreatynowy hydrolizat kazeiny 15g, papainowy hydrolizat maczki sojowej 5g, chlorek sodu 4g, L-cystyna 0,7g, siarczan (IV) sodu 0,2g
Neutralizer : *Broth Eugon LT 100: composition: pancreatin casein hydrolyzate 15g, papain soybean poppy hydrolyzate 5g, sodium chloride 4g, L-cystine 0.7g, sodium sulfate (IV) 0.2g*

Pożywki mikrobiologiczne : ***Media :*** Bacteria - Tryptic soy agar (TSA) ; drożdże i pleśnie/yest & mulds - Sabouraud Dextrose Agar medium (SDA) ;

Warunki inkubacji : 32,5 ± $2,5^0$ C; 3 - 5 days (bacteria)
Incubation conditions : 22,5 ± $2,5^0$ C; 3 - 5 days (yeast & mulds)

Wielkość próbki : 20g [19,5 - 20,4 g] próbki dla każdego szczepu wzorcowego, 22,5 ± 2,5ºC, 28 dni
Sample size : *20g [19.5-20.4 g] samples for each reference strain, 22.5 ± 2.5ºC, 28 days*

Kalibrowana zawiesina : 0,2 ml kalibrowanej zawiesiny, stężenie końcowe w próbce: 1,0 x 10^5 - 1,0 x 10^6 CFU/g
Calibrated suspension: *0.2 ml of calibrated suspension, final concentration in the sample: 1.0 x 10^5 - 1.0 x 10^6 CFU / g*

L.p. *No.*	Parametr *Parameter*	szczep *strain*	Nvf [CFU/g]	Nvn [CFU/g]	Nvf ≥ 0,5 Nvn	Kontrola Nv *Controle Nv*	N [CFU/ml]	No [CFU/ml]	(3)
1.	**Wykazanie skuteczności neutralizatora**<br>***Demonstration of neutralizer effectiveness***<br>Nvf - liczba jtk w mieszaninie neutralizatora i badanej próbki<br>*No. of CFU in the mixture with neutralizer and tested sample*<br>Nvn - liczba jtk mieszaninie bez badanej próbki<br>*No. of CFU in the mixture without tested sample*<br>Nvf ≥ 0,5 Nvn<br>**Inokulum /** ***Inoculum***<br>N - początkowa liczba drobnoustrojów<br>*initial number of microorganisms*<br>No - liczba drobnoustrojów po czasie To<br>*number of microorganisms after time To*<br>No=N/100	*Escherichia coli* ATCC 8739	**5,3 x 10^1**	**5,6 x 10^1**	**≥ 0,5**	**5,2 x 10^1**	**5,3 x 10^6**	**5,3 x 10^5**	(N)
		Staphylococcus aures ATCC 6538	**4,1 x 10^1**	**4,5 x 10^1**	**≥ 0,5**	**3,9 x 10^1**	**4,1 x 10^6**	**4,1 x 10^5**	
		Pseudomonas aeruginosa ATCC 9027	**3,8 x 10^1**	**4,0 x 10^1**	**≥ 0,5**	**3,7 x 10^1**	**3,7 x 10^6**	**3,7 x 10^5**	
		Candida albicans ATCC 10231	**3,6 x 10^1**	**3,7 x 10^1**	**≥ 0,5**	**3,0 x 10^1**	**3,2 x 10^6**	**3,2 x 10^5**	
		Aspergillus brasiliensis ATCC 16404	**4,3 x 10^1**	**4,5 x 10^1**	**≥ 0,5**	**4,0 x 10^1**	**4,1 x 10^6**	**4,1 x 10^5**	

WYNIKI / *RESULTS*

L.p. *No.*	Parametr *Parameter*	szczep *strain*	Logarytm redukcji w czasie Tx [Rx] / *Logarithm of reduction in time Tx [Rx]* T2	kryterium *criterion*	T7	kryterium *criterion*	T14	kryterium *criterion*	T28	kryterium *criterion*	(3)
2.	**Test konserwacji**<br>***Challenge test***<br>Rx = lg No - lg Nx<br>No - liczba drobnoustrojów w czasie To<br>*No. of CFU at time To*<br>Nx - liczba drobnoustrojów w czasie Tx<br>*No. of CFU at time Tx*<br>Nl - liczba zdolnych do życia CFU nie zwiększa się w porównaniu z poprzednim odczytem<br>*No. of CFU does not increase to previous reading*<br>Rx = 0 gdy / when lg No = lg Nx<br>(brak wzrostu populacji względem wyjściowego zliczania) / *(no population increase relative to the initial count)*	*Escherichia coli* ATCC 8739	**4,25**	**≥ 2**	**4,25**	**≥ 3**			**4,25 Nl**	**≥ 3 i Nl**	(N)
		Staphylococcus aures ATCC 6538	**4,14**	**≥ 2**	**4,14**	**≥ 3**			**4,14 Nl**	**≥ 3 i Nl**	
		Pseudomonas aeruginosa ATCC 9027	**4,09**	**≥ 2**	**4,09**	**≥ 3**			**4,09 Nl**	**≥ 3 i Nl**	
		Candida albicans ATCC 10231					**4,03**	**≥ 2**	**4,03 Nl**	**≥ 2 i Nl**	
		Aspergillus brasiliensis ATCC 16404					**4,44**	**≥ 2**	**4,44 Nl**	**≥ 2 i Nl**	

Zgodnie z Ph. Eur. 10. tab. 5.1.3-2 preparaty do uszu, preparaty do nosa, preparaty do stosowania na skórę i preparaty do inhalacji
According to the EP 10.0, tab. 5.1.3-2 for ear preparations, nasal preparations, preparations for cutaneous application and preparations for inhalation

Wymagania / *Requirements*								
**mikroorganizmy /** *microorganisms*	**Bakterie /** *Bacteria*				**Grzyby /** *Moulds*			
**czas pobrania próbki /** *sampling time*	**T2**	**T7**	**T14**	**T28**	**T2**	**T7**	**T14**	**T28**
**Kryterium A /** *Criterion A*	≥ 2	≥ 3	-	≥ 3 i Nl	-	-	≥ 2	≥ 2 i Nl
**Kryterium B /** *Criterion B*	-	-	≥ 3	≥ 3 i Nl	-	-	≥ 1	≥ 1 i Nl

Legenda:

(1) - zgodnie z wewnętrznymi wymaganiami Klienta
(2) - badanie podzlecone
(3) - badanie/pobór próbki oznaczone symbolem (A) - badanie/pobór próbki planowane do akredytacji w 2020 roku; symbolem (N) - badanie/pobór próbki nieplanowane do akredytacji w 2020 roku
N/A - nie dotyczy

Legend:

(1) - according to internal Customer requirements
(2) - sub-contracted test
(3) - parameter (A) method / sampling planned for accreditation in 2020 ; parameter (N) - method / sampling not planned for accreditation in 2020
N/A - not applicable

UWAGI: Uzupełnienie do Sprawozdania z badań nr: 0001 - MB / A / 09 / 20, rev. 02. Błąd w kryterium akceptacji dla P. aeruginosa w T2, dostosowanie tabeli z kryteriami do wymagań Klienta.
COMMENTS: Supplement to the Test Report no. 0001 - MB / A / 09 / 20, rev. 02. Error in criterion for P. aeruginosa at T2, adjusting the table with the criteria to the client's requiremen.

**Autoryzował :** *Authorized :*	30.10.2020	drNowaczyk: 003		Kierownik Pracowni Mikrobiologii i Fizyko-Chemii (kwalifikowany podpis elektroniczny)* *Microbiological & Physico-Chemical Laboratory Manager (qualified electronic signature)**
	data / *date*	Nr pracownika / *Employee no*	kod QR /*QR code*	funkcja / *position*

UWAGA : Bezpieczny podpis elektroniczny weryfikowany przy pomocy certyfikatu kwalifikowanego jest równoważny pod względem skutków prawnych z podpisem własnoręcznym. Oryginalene sprawozdanie z badań są wydawane w formie elektronicznej z rozszerzeniem "*-sig.pdf" lub "*_signed.pdf". W związku z tym wszystkie wydruki, o ile nie są potwierdzone za zgodność z oryginałem, są kopiami.
COMMENTS : Secure electronic signature verified by a qualified certificate is equivalent in terms of legal consequences handwritten signature. The original test report is issued in electronic form with the extension " -sig.pdf" lub "*-signed.pdf". Therefore, all prints, if not confirmed as being true to the original, are copies.*

Badania wykonywane w dr Nowaczyk Centrum Badań i Innowacji Sp. z o.o.Sp.K. są prowadzone zgodnie ze standardowymi metodami badawczymi. Uzyskane wyniki są zgodne z przyjętymi międzynarodowymi standardami, chyba że zostały ustalone inne szczegółowe wymagania, zatwierdzone na piśmie przez dr Nowaczyk Centrum Badań i Innowacji Sp. z o.o.Sp.K. oraz Klienta, przed rozpoczęciem badania. Wyniki badań odnoszą się wyłącznie do przebadanych próbek, w określonych warunkach i przy użyciu metod badawczych i procedur uzgodnionych wcześniej z Klientem. Obowiazkiem Klienta jest zapewnienie, że każda jednostka produktu i opakowania jest wyprodukowana w sposób identyczny. dr Nowaczyk Centrum Badań i Innowacji Sp. z o.o.Sp.K. nie ponosi odpowiedzialności za jakiekolwiek wnioski, interpretacje i uogólnienia wykonane przez Klienta z wydanych przez dr Nowaczyk Centrum Badań i Innowacji Sp.z o.o.Sp.K. sprawozdań z badań. Niniejsze Sprawozdanie z Badania jest poufną własnością Klienta. Bez pisemnej zgody dr Nowaczyk Centrum Badań i Innowacji Sp. z o.o.Sp.K. sprawozdanie z badania nie może być powielane inaczej jak tylko w całości.

Testing at dr Nowaczyk Centrum Badań i Innowacji Sp.z o.o.Sp.K. is performed according to standard test procedures. Reported test results are accurate to generally accepted international standards, unless specific requirements have been agreed to in writing by dr Nowaczyk Centrum Badań i Innowacji Sp.z o.o.Sp.K. and Customer prior to the test commencing. Our reports apply only to the specific samples tested, under stated conditions and using test methods and routines agreed in advance with our customers. It is the manufacturer's responsibility to assure that additional production units of product and package are manufactured identically. dr Nowaczyk Centrum Badań i Innowacji Sp.z o.o.Sp.K. shall have no liability for any deductions, inferences of generalisations drawn by the Customers or others from dr Nowaczyk Centrum Badań i Innowacji's issued reports. This report is the confidential property of dr Nowaczyk Centrum Badań i Innowacji Sp.z o.o.Sp.K. and its Customer. Extracts from the test report shall not be reproduced, except in full, and not without prior written approval of dr Nowaczyk Centrum Badań i Innowacji Sp.z o.o.Sp.K.

Sprawozdanie z badania zostało sporządzone w jednym egzemplarzu.	Oryginał nr 1 :	Klient	Kopia egzemplarza nr 1 :	Archiwum
Test Report prepared in single copy.	*Oryginal copy No. 1 :*	*Customer*	*Duplicate of copy No. 1 :*	*Archive*
