# Supplementary material for: Zinc(II) Complexes of Amino Acids as New Active Ingredients for Anti-Acne Dermatological Preparations
Source: Int J Mol Sci. 2021 Feb 6;22(4):1641. doi: 10.3390/ijms22041641 (PMC7915519; doi:10.3390/ijms22041641)
Supplement: Supplementary file 1 [file ijms-22-01641-s001.zip › ijms-1083706 suppl/Test report S8 - dermatological test (Patch test) for ZnHis gel (B).pdf]

dr Nowaczyk Centrum Badań i Innowacji Sp. z o.o. Sp.K.
ul. Żmigrodzka 81-83, lok. 205, 51-130 Wrocław, Poland
tel. +48 509 387 307, e-mail: biuro@drnowaczyk.com
www.drnowaczyk.com

Laboratorium badawcze zgodne z normą ISO / IEC 17025

Testing laboratory according to the standard ISO / IEC 17025

Sprawozdanie z badań, nr :
Test report, no : **0014 - DR / A / 09 / 20 rev. 01**
(zgodnie z / acc. to LAB-05.00.03, rev.02)

BADANIE DERMATOLOGICZNE / KOSMETYKI / *DERMATOLOGICAL TEST / COSMETICS*

**Zleceniodawca :** *Customer :*	**Zakład Chemii Surowców Kosmetycznych Uniwersytet Medyczny w Łodzi ul. Muszyńskiego 1 91-151 Łódź**	**Kontrahent.:** *Contractor :*	**Zakład Chemii Surowców Kosmetycznych Uniwersytet Medyczny w Łodzi ul. Muszyńskiego 1 91-151 Łódź**
**Nazwa próbki:** *Name of sample:*	**żel ŻDD_Zn-His_2.5%_1_16.07.20** *Gel ŻDD_Zn-His_2.5%_1_16.07.20*		
**Numer zlecenia:** *Protocol-order No.:*	0014 – Z / 09 / 20	**Metoda pobrania próbki (5) :** *Method of sampling (5):*	Nie podano *Not given*
**Próbka pobrane przez:** *Sample collected by:*	Klienta *Customer*	**Postępowanie z próbką wg:** *Handling of sample acc to:*	LAB-05.00.00, rev. 02
**Stan i ilość próbki:** *Sample evaluation & quantity:*	Bez zastrzeżeń *Correct*	**Data przyjęcia próbki:** *Date of receiving sample:*	02.09.2020
**Numer próbki:** *Sample No.:*	0058 – P / 09 / 20	**Data rozpoczęcia badania:** *Date of analysis start:*	19.10.2020
**Numer analizy :** *Analysis No.:*	0014– DR / A / 09 / 20	**Data zakończenie badania:** *Date of of analysis end:*	23.10.2020

Metoda badawcza (5): *Test method (5):*
MD-0xx.0, rev.01 „Badanie dermatologiczne testem płatkowym półotwartym"
MD-0xx.00, rev. 01 „Badanie dermatologiczne testem płatkowym otwartym"
MD-0XX.00, rev.01 „Dermatological semi-open patch test"
MD-0xx.00, rev.01 „Dermatological open patch test"

Metoda badań zgodna z: *Method of test acc to:*

- Rozporządzenie Parlamentu Europejskiego i Rady (WE) nr 1223/2009 z dnia 30 listopada 2009 r. dotyczące produktów kosmetycznych (Dz. Urz. UE L 342 z 22.12.2009);
  Regulation No 1223/2009 of the European Parliament and of the Council of 30 November 2009 on cosmetic products (OJ L 342, 22.12.2009);
- Decyzja Wykonawcza Komisji z dnia 25 listopada 201 r. w sprawie wytycznych dotyczących załącznika I do rozporządzenia Parlamentu Europejskiego i Rady (WE) nr 1223/2009 dotyczącego produktów kosmetycznych;
  Commission Implementing Decision of 25 November 2013 on Guidelines on Annex I to Regulation (EC) No 1223/2009 of the European Parliament and of the Council on cosmetic products;
- Ustawa z dnia 4 października 2018 r. o produktach kosmetycznych (Dz. U. 2018, poz. 2227);
  Act on cosmetic products of 4 October 2018 (Journal of Laws of 2018, item 2227);
- Cosmetics Europe – The Personal Care Association Guidelines „Product Test Guidelines for the Assessment of Human Skin Compatibility 1997";
  Cosmetics Europe – The Personal Care Association Guidelines "Product Test Guidelines for the Assessment of Human Skin Compatibility 1997;"
- Cosmetics Europe – The Personal Care Association Guidelines „Guidelines for the Assessment of Skin Tolerance of Potentially Irritant Cosmetic Ingredients, Edition of 1997";
  Cosmetics Europe – The Personal Care Association Guidelines "Guidelines for the Assessment of Skin Tolerance of Potentially Irritant Cosmetic Ingredients, Edition of 1997;"
- Cosmetics Europe - The Personal Care Association Guidelines for the Evaluation of the Efficacy of Cosmetic Products 2008
- The SCCS's Notes of Guidance for the Testing of Cosmetic Substances and their Safety Evaluation 10th Revision – SCCS/1602/18 Final version
- „Basic Criteria of The protocols for the skin compatibility testing of potentially cutaneous irritant cosmetic ingredients of mixtures of ingredients on human volunteers" – SCCNFP/0245/99 Final December 1999
- Deklaracja Helsińska z 1964 r. wraz z późniejszymi zmianami
  Declaration of Helsinki of 1964 as amended;
- „Memorandum on use of Human Data in risk assessment of skin sensitisation" – SCCS/1567/15

Wyniki badania odnoszą się wyłącznie do badanego obiektu
Results are connected only with samples which were analysed

Spis treści / *Index*

1. Podstawa wykonania badania / *Study basis* ... 2
2. Charakterystyka próbki / *Sample characteristics* ... 2
3. Opis metody / *Description of the method* ... 3
4. Cel badania / *Purpose of the study* ... 3
5. Dobór probantów do badania / *Selection of subjects* ... 3
6. Wyniki badania / *Test result* ... 4
7. Wnioski / *Conclusion* ... 4
8. Zatwierdzenie / *Approval* ... 5

1. Podstawa wykonania badania / *Study basis*

- Próbka do badań dostarczona przez Zleceniodawcę
  Test sample delivered by the Customer
- Skład jakościowy próbki dostarczony przez Zleceniodawcę
  Test sample qualitative composition delivered by the Customer
- Wyniki czystości mikrobiologicznej próbki dostarczone przez Zleceniodawcę
  Test sample microbiological purity results delivered by the Client
- Informacje o sposobie użycia i przeznaczeniu produktu
  Information about the manner of use and indications of the product

2. Charakterystyka próbki / *Sample characteristics*

Zgodnie ze specyfikacją jakościową producenta:
Acc. to the manufacturer's quality Specification:

Wygląd produktu: *Product appearance:*	Żel *Gel*
Barwa: *Color:*	Jasno słomkowa *Light straw*
Zapach: *Smell:*	Charakterystyczny dla użytych surowców *Typical of the materials used*
Opakowanie: *Package:*	Opakowanie zastępcze zawierające nazwę próbki *Replacement packaging with the sample name*
Przeznaczenie produktu: *Product designation:*	Żel do skóry trądzikowej *Gel for acne skin*
INCI (*) :	Aqua, Carrageenan, Zinc Histidine, Phenoxyethanol, Ethylhexylglycerin, Citric Acid.

(*) - sprawozdanie nie zawiera analizy składu próbki; za zgodność przygotowanych do badań próbek z deklarowanym składem jakościowym i czystością mikrobiologiczną produktu odpowiada Zleceniodawca / *The report does not include sample composition analysis; the Client is responsible for the consistency of the test samples with the declared qualitative composition and microbiological purity of the product*

3. Opis metody / *Description of the method*

Testy płatkowe wg Jadassohna-Blocha (w modyfikacji Rudzkiego) prowadzono pod nadzorem lekarza dermatologa. Ocenę właściwości alergizujących i podrażniających preparatu przeprowadzono na grupie 25 zdrowych ochotników z wywiadem alergologicznym (panel wrażliwy) / ~~bez wywiadu alergologicznego (panel zwykły)~~ .
(niepotrzebne skreślić)

Patch tests according to Jadassohn-Bloch (as modified by Rudzki) were performed under the supervision of a dermatologist. The evaluation of sensitising and irritant properties of the product was made based on a sample of 25 healthy volunteers with (sensitive panel) / ~~without (normal panel)~~ a history of allergy.
(delete as appropriate)

Badanie przeprowadzono przy zastosowaniu plastrów Finn Chamber z bibułkami filtracyjnymi, na które nanosi się badany produkt, a następnie całość przykleja na skórę ochotnika (ramię lub okolica międzyłopatkowa). Próby zdejmowano po 48 h. Pierwszego odczytu dokonano po 15-20 minutach od zdjęcia plastra, a następie po 72 h.
The study was conducted using Finn Chamber patches with filter paper onto which the tested product was applied; the whole patch was then applied to the volunteer's skin (arm or interscapular region). The patches were removed after 48 h. The first reading was done 15–20 minutes after patch removal, and subsequently after 72 h.

Odczyt został wykonany wg ogólnie przyjętej skali w badaniach dermatologicznych, a następnie oceniony przez lekarza dermatologa.
The reading was based on a generally acknowledged scale for dermatological studies, and subsequently evaluated by a dermatologist.

Odczyn dodatni (rumień) potwierdza alergizujące właściwości preparatu, zaś wynik ujemny (brak rumienia) potwierdza brak właściwości alergizujących.
A positive reaction (erythema) confirms sensitising properties, while a negative reaction (no erythema) confirms the lack of sensitising properties.

4. Cel badania / *Purpose of the study*

Ocena właściwości drażniących / uczulających produktu u zdrowego, dorosłego ochotnika metodą jednokrotnego nałożenia testu płatkowego.
Assessment of irritant / sensitising properties of the product in a healthy adult volunteer with a single application of a patch test.

5. Dobór probantów do badania / *Selection of subjects*

Dobór ochotników uwzględnia kryteria włączeń i wyłączeń do badań.
The volunteers were selected on the basis of inclusion and exclusion criteria.

Główne kryteria włączenia: zdrowe kobiety i mężczyźni w wieku 18-65 lat, fototyp: I – IV w skali Fitzpatrick'a, typ skóry Kaukaski.
The main inclusion criteria were: healthy females and males aged 18–65 years; phenotype: I–IV in the Fitzpatrick scale, Caucasian skin type.

W badaniu wzięło udział 25 ochotników, którzy wyrazili dobrowolną zgodę na udział w badaniu.
The study involved 25 volunteers who expressed a voluntary consent to the participation in the study.

Skóra w miejscu badania była prawidłowa, bez podrażnień i zmian chorobowych.
The skin at the test site was normal, without irritation or pathological changes.

Ochotników poinformowano o sposobie szczególnej ostrożności w postępowaniu z testem płatkowym.

The volunteers were informed about the manners of particular caution with respect to patches.

6. Wyniki badania / *Test result*

Tab. 1. Skala oceny wyników badań zgodnie z zaleceniami Międzynarodowej Grupy Badającej Wyprysk Kontaktowy (ICDRG).
The assessment scale was consistent with the recommendations of the International Contact Dermatitis Research Group (ICDRG)

**Skala oceny** *Assessment scale*	**Wynik** *Result*	**Typ odczynu** *Reaction type*
(-)	Odczyn ujemny *Negative reaction*	Brak zmian skórnych *No skin changes*
(?)	Odczyn wątpliwy / podrażnienie od plastra *Doubtful result / patch irritation*	Lekki odczyn rumieniowy *Slight erythema*
(+)	Słaby odczyn *Mild reaction*	Odczyn rumieniowy, wyczuwalny palpacyjnie, bez pęcherzyków *Palpable erythema, no vesicles*
(++)	Silny odczyn *Strong reaction*	Odczyn rumieniowy, grudki, obecne pęcherzyki lub obrzęk *Erythema, papules, vesicles or swelling*
(+++)	Bardzo silny odczyn *Very strong reaction*	Odczyn rumieniowy, grudki, pęcherzyki i /lub owrzodzenia Erythema, papules, vesicles and/or ulceration
(IR)	Reakcja podrażnieniowa *Irritant reaction*	Stan zapalny, brak nacieku, krostki, rumień, tendencja do wygasania *Inflammation, no infiltration, papules, erythema, tendency to subside*

Tab. 2. Wyniki badania / *Test results*

**Ochotnik** *Volunteer*	**Wiek** *Age*	**Płeć*** *Sex**	**Fototyp skóry** *Skin phototype*	**Miejsce aplikacji** *Application area*	**Wynik** *Result*	**Ochotnik** *Volunteer*	**Wiek** *Age*	**Płeć*** *Sex**	**Fototyp skóry** *Skin phototype*	**Miejsce aplikacji** *Application area*	**Wynik** *Result*
1.	36	K/F	II	Arm	(-)	14.	36	K/F	III	Arm	(-)
2.	42	K/F	I	Arm	(-)	15.	28	K/F	II	Arm	(-)
3.	40	K/F	III	Arm	(-)	16.	42	K/F	II	Arm	(-)
4.	32	K/F	III	Arm	(-)	17.	35	K/F	I	Arm	(-)
5.	60	K/F	I	Arm	(-)	18.	44	K/F	II	Arm	(-)
6.	44	K/F	II	Arm	(-)	19.	56	K/F	II	Arm	(-)
7.	46	K/F	III	Arm	(-)	20.	53	K/F	III	Arm	(-)
8.	43	K/F	II	Arm	(-)	21.	31	K/F	II	Arm	(-)
9.	39	K/F	II	Arm	(-)	22.	30	K/F	II	Arm	(-)
10.	55	K/F	III	Arm	(-)	23.	33	K/F	I	Arm	(-)
11.	38	K/F	II	Arm	(-)	24.	30	K/F	II	Arm	(-)
12.	39	K/F	I	Arm	(-)	25.	40	K/F	II	Arm	(-)
13.	30	K/F	II	Arm	(-)						

* - K/F – kobieta / *female* / M/M – mężczyzna / *male*

7. Wnioski / *Conclusions*

Na podstawie przeprowadzonego testu płatkowego stwierdza się, że badany produkt:

żel ŻDD_Zn-His_2.5%_1_16.07.20

nie działa / ~~działa~~ drażniąco ani / ~~oraz~~ alergizująco na skórę badanych osób.
(niepotrzebne skreślić) (niepotrzebne skreślić)

Based on the patch test, it has been shown that the tested product:

Gel ŻDD_Zn-His_2.5%_1_16.07.20

has no / ~~has~~ irritant or / ~~and~~ sensitising effects on the skin of the subjects.

(delete as appropriate) *(delete as appropriate)*

Wydana opinia nie dotyczy osób, u których występuje udokumentowana alergia na którykolwiek składnik tego produktu.
The issued opinion does not refer to persons with a documented allergy to any of the product's components.

8. Zatwierdzenie / *Approval*

**Opracował :** ***Prepared :***	26/10/2020	drNowaczyk: 001	Dyrektor Laboratorium *Laboratory Director*	(kwalifikowany podpis elektroniczny) *(qualified electronic signature)*
	data / *date*	Nr pracownika / *Employee no*	funkcja / *position*	
**Autoryzował :** ***Authorized :***	26/10/2020	drNowaczyk: 005	Specjalista dermatolog - wenerolog *Specialist dermatologist - venerologist*	
	data / *date*	Nr pracownika / *Employee no*	funkcja / *position*	

UWAGA : Bezpieczny podpis elektroniczny weryfikowany przy pomocy certyfikatu kwalifikowanego jest równoważny pod względem skutków prawnych z podpisem własnoręcznym. Oryginalne sprawozdanie z badań są wydawane w formie elektronicznej z rozszerzeniem "*-sig.pdf" lub "*_signed.pdf". W związku z tym wszystkie wydruki, o ile nie są potwierdzone za zgodność z oryginałem, są kopiami.

COMMENTS : *Secure electronic signature verified by a qualified certificate is equivalent in terms of legal consequences handwritten signature. The original test report is issued in electronic form with the extension "* -sig.pdf" lub "*-signed.pdf". Therefore, all prints, if not confirmed as being true to the original, are copies.*

Badania wykonywane w dr Nowaczyk Centrum Badań i Innowacji Sp. z o.o.Sp.K. są prowadzone zgodnie ze standardowymi metodami badawczymi. Uzyskane wyniki są zgodne z przyjętymi międzynarodowymi standardami, chyba że zostały ustalone inne szczegółowe wymagania, zatwierdzone na piśmie przez dr Nowaczyk Centrum Badań i Innowacji Sp. z o.o.Sp.K. oraz Klienta, przed rozpoczęciem badania. Wyniki badań odnoszą się wyłącznie do przebadanych próbek, w określonych warunkach i przy użyciu metod badawczych i procedur uzgodnionych wcześniej z Klientem. Obowiązkiem Klienta jest zapewnienie, że każda jednostka produktu i opakowania jest wyprodukowana w sposób identyczny. dr Nowaczyk Centrum Badań i Innowacji Sp. z o.o.Sp.K. nie ponosi odpowiedzialności za jakiekolwiek wnioski, interpretacje i uogólnienia wykonane przez Klienta z wydanych przez dr Nowaczyk Centrum Badań i Innowacji Sp.z o.o.Sp.K. sprawozdań z badań. Niniejsze Sprawozdanie z Badania jest poufną własnością Klienta. Bez pisemnej zgody dr Nowaczyk Centrum Badań i Innowacji Sp. z o.o.Sp.K. sprawozdanie z badania nie może być powielane inaczej jak tylko w całości. Odpowiedzialność dr Nowaczyk Centrum Badań i Innowacji Sp. z o.o.Sp.K. jest ograniczona wyłącznie do danych zawartych w oryginale sprawozdania.

The studies performed in Dr Nowaczyk Research and Innovation Centre Sp. z o.o. Sp.K. are conducted in accordance with standard research methodology. The results are concordant with acknowledged international standards unless other specific requirements have been established and approved in writing by Dr Nowaczyk Research and Innovation Centre Sp. z o.o. Sp.K. and the Client prior to the initiation of the study. The study results refer solely to the tested samples, given conditions and adopted research methods and procedures agreed on previously with the Client. It is the Client's obligation to produce each unit of the product and each package in an identical way. Dr Nowaczyk Research and Innovation Centre Sp. z o.o. Sp.K. is not responsible for any conclusions, interpretations and generalisations made by the Client based on the study reports issued by Dr Nowaczyk Research and Innovation Centre Sp. z o.o. Sp.K. This Study Report is confidential Client's property. Without the Client's consent in writing, Dr Nowaczyk Research and Innovation Centre Sp. z o.o. Sp.K. shall not copy the study report in other ways than in whole. The liability of Dr Nowaczyk Research and Innovation Centre Sp. z o.o. Sp.K. is restricted solely to the data included in the original report.

Sprawozdanie z badania zostało sporządzone w jednym egzemplarzu. *Test Report prepared in single copy.*	Oryginał nr 1 : Klient *Oryginal copy No. 1 : Customer*	Kopia egzemplarza nr 1 : Archiwum *Duplicate of copy No. 1 : Archive*
